# New statistical selection method for pleiotropic variants associated with both quantitative and qualitative traits

**DOI:** 10.1186/s12859-023-05505-8

**Published:** 2023-10-10

**Authors:** Kipoong Kim, Tae-Hwan Jun, Bo-Keun Ha, Shuang Wang, Hokeun Sun

**Affiliations:** 1 Department of Statistic, Pusan National University, 46241 Busan, Korea; 2 Department of Plant Bioscience, Pusan National University, 50463 Miryang, Korea; 3 Department of Applied Plant Science, Chonnam National University, 61186 Gwangju, Korea; 4 Department of Biostatistics, Mailman School of Public Health, Columbia University, New York, 10032 USA

**Keywords:** Pleiotropic variants, Genetic association, Multiple phenotypes, Regularization, Selection probability

## Abstract

**Background:**

Identification of pleiotropic variants associated with multiple phenotypic traits has received increasing attention in genetic association studies. Overlapping genetic associations from multiple traits help to detect weak genetic associations missed by single-trait analyses. Many statistical methods were developed to identify pleiotropic variants with most of them being limited to quantitative traits when pleiotropic effects on both quantitative and qualitative traits have been observed. This is a statistically challenging problem because there does not exist an appropriate multivariate distribution to model both quantitative and qualitative data together. Alternatively, meta-analysis methods can be applied, which basically integrate summary statistics of individual variants associated with either a quantitative or a qualitative trait without accounting for correlations among genetic variants.

**Results:**

We propose a new statistical selection method based on a unified selection score quantifying how a genetic variant, i.e., a pleiotropic variant associates with both quantitative and qualitative traits. In our extensive simulation studies where various types of pleiotropic effects on both quantitative and qualitative traits were considered, we demonstrated that the proposed method outperforms the existing meta-analysis methods in terms of true positive selection. We also applied the proposed method to a peanut dataset with 6 quantitative and 2 qualitative traits, and a cowpea dataset with 2 quantitative and 6 qualitative traits. We were able to detect some potentially pleiotropic variants missed by the existing methods in both analyses.

**Conclusions:**

The proposed method is able to locate pleiotropic variants associated with both quantitative and qualitative traits. It has been implemented into an R package ‘UNISS’, which can be downloaded from http://github.com/statpng/uniss.

**Supplementary Information:**

The online version contains supplementary material available at 10.1186/s12859-023-05505-8.

## Background

In the last few decades, genome-wide association studies (GWASs) have successfully discovered a number of genetic variants associated with various phenotypes of interest, providing an unprecedented opportunity for researchers to explore the genetic architecture underlying complex traits and diseases. Recent years, there is renewed attention to genetic pleiotropy which refers to the phenomenon where a single genetic variant is associated with multiple phenotypes. In different organisms, pleiotropic variants have been studied to understand a mechanistic underpinning of multiple traits. For example, a majority of patients with cystic fibrosis are also infertile due to congenitial bilateral absence of vas deferens (CBAVD). This is an evidence that a mutation in CFTR gene plays a shared role in both lung function and CBAVD [1]. Another example is the frizzled feather trait, which is a well known pleiotropy in chickens [2]. Recently, a pleiotropic effect on hyperpigmentation and egg production was discovered by chicken genome analysis [3]. In plant biology, the recent advent of high-throughput plant phenotyping technologies also contributed to the attention given to pleiotropy, which plays a potential role in improvement of the genetic gain in yields of major crops such as wheat (*Triticum aestivum L.*) stagnated over decades [4].

Identification of pleiotropic variants may address the missing heritability problem caused by genetic variants that cannot be detected by single-trait GWAS [5]. For example, recent studies have successfully identified pleiotropic variants using multi-phenotype association models which can increase the statistical power of detecting SNPs [6, 7]. They demonstrated that genetic overlap between multiple traits can boost the power of weak association signals that cannot be detected by single-trait analyses. Additional investigations found that 4.6% of SNPs and 16.9% of genes listed in the National Human Genome Research Institute (NHGRI) catalog could be associated with multiple phenotypes [8]. Also, it suggests that 90% of trait-associated genetic variants could influence multiple phenotypes through analyzing 4,155 publicly available GWAS results across 2,965 unique traits [9]. Therefore, studies on genetic pleiotropy are expected to advance genetic association studies which have mostly focused on single-trait analyses.

Many statistical methods have been proposed to identify pleiotropic variants with majority of them based on multivariate approaches. For instance, dimension reduction techniques such as principal component analysis [10] and canonical correlation analysis [11] were applied to multivariate traits. Both methods essentially extract weighted linear combinations of multiple quantitative trait values. One of the most straightforward methods is to calculate individual *p*-values one for association of one genetic variant on one phenotype with adjustment for multiple testing [12]. However, it has low power to detect weak association signals due to its conservative adjustment. Multivariate analysis of variance (MANOVA) tests were also used to detect pleiotropic variants, assuming a multivariate normal distribution of multiple traits [13, 14]. Also, a generalized estimating equation (GEE) [15], a generalized linear mixed model [16] and a multivariate linear mixed model controlling for population structure [17] have been proposed. These methods consider multiple traits as repeated measurements of each individual in order to account for correlations among multiple traits. However, they cannot be applied to mixed quantitative and qualitative traits. This is a statistically challenging problem because there does not exist an appropriate multivariate distribution to model both quantitative and qualitative data together [15].

There have been several attempts to detect pleiotropic variants associated with mixed quantitative and qualitative traits. The extended GEE for bivariate traits was proposed by Liu et al. [18], combining two different generalized linear models one for a continuous trait and one for a binary trait. Similarly, Schaid et al. [19] applied GEE to the standardized residuals extracted from separate generalized linear models for each trait. O’Reilly et al. [20] suggested to use a reverse regression, where a single variant is regarded as a dependent variable while multiple traits such as quantitative and qualitative traits are considered as independent variables. Alternatively, meta-analysis methods which integrate summary statistics of individual variants associated with either a quantitative or a qualitative trait can be applied. Recently, many researches have suggested meta-analysis methods for detecting multiple phenotype associations [21–25]. However, these existing methods developed for mixed quantitative and qualitative traits cannot account for correlations among genetic variants because genetic association of multiple traits are tested on individual variants one at a time.

Regularization methods based on penalized regression have been popularly applied to analysis of high-dimensional genomic data [26–33]. Since multiple genetic effects on a phenotype outcome are simultaneously estimated in one regression framework, regularization methods are able to consider genetic correlations among variants. However, most of regularization methods are designed for a single phenotype outcome, including quantitative and qualitative traits. Although there exist some regularization methods for a multivariate response [34–36], they are all limited to only quantitative traits.

In this article, we propose a new statistical selection method based on a ${\underline{\textrm{uni}}}$fied ${\underline{\textrm{s}}}$election ${\underline{\textrm{s}}}$core (UNISS), which linearly combines the selection probability of individual variants over multiple traits. The selection probability quantifies the strength of association between genetic variants and a phenotype outcome. Therefore, the unified selection scores of genetic variants associated with either a quantitative or qualitative trait tend to be high, while the scores of variants with no association are relatively low. With an appropriate cut off of the unified selection scores, we can select a certain number of pleiotropic variants. In the next section, we described our statistical selection method, including how to pick up the cut-off of the unified selection score. In the section of Results we presented simulation results, comparing selection performance of the proposed UNISS to that of several competing methods under various simulation scenarios of pleiotropic effects on both quantitative and qualitative traits. Continuously, we also presented plant SNPs data application on two different datasets, one with 6 quantitative and 2 qualitative traits from 235 peanut accessions, and one with 2 quantitative and 6 qualitative traits from 354 cowpea accessions. Lastly, we summarized the methods and discussed future directions.

## Materials and methods

### Elastic-net regularization and selection probability

Suppose that we observe *p* genetic variants and *q* phenotypes from *n* individuals. We then denote the dataset of the *i*th individual by $({\varvec{x}}_{i}, {\varvec{y}}_{i})$ for $i=1, \ldots , n$, where ${\varvec{x}}_i = (x_{i1}, \ldots , x_{ip})^{{\text{T}}}$ is the *p*-dimensional vector of genetic values and ${\varvec{y}}_{i} = (y_{i1}, \ldots , y_{iq})^{{\text{T}}}$ is the *q*-dimensional vector of phenotypic outcomes. A regression coefficient matrix is denoted by $\varvec{\beta }=(\varvec{\beta }_1,\ldots ,\varvec{\beta }_q)$, where $\varvec{\beta }_k=(\beta _{0k},\beta _{1k},\ldots ,\beta _{pk})^{{\text{T}}}$. Then, the penalized likelihood of the *k*th phenotype outcome for the elastic-net regularization [37] is defined as

$$\begin{aligned} \sum _{i=1}^n \left( {\varvec{y}}_{ik}-\beta _{0k}-{\varvec{x}}_i^{{\text{T}}}\varvec{\beta }_k\right) ^2 + \lambda \left( \alpha \sum _{j=1}^p |\beta _{jk}| + (1-\alpha )\sum _{j=1}^p \beta _{jk}^2\right) , \end{aligned}$$

if the *k*th phenotype outcome is quantitative. Note that the penalized likelihood consists of a least square loss function and a elastic-net penalty function. The elastic-net uses two tuning parameters $\lambda$ and $\alpha$ in the penalty function. $\lambda > 0$ controls the number of nonzero regression coefficients, so all coefficients can be exactly zero if $\lambda$ is relatively large. $\alpha \in [0,1]$ is a mixing proportion between lasso regularization and ridge regularization. Friedman et al. [38] developed a cyclic coordinate descent algorithm to estimate the regression coefficients of elastic-net regularization for fixed values of $\alpha$ and $\lambda$. They also suggested to use cross-validation for estimation of the tuning parameters. In general, a small value of $\alpha$ is desirable for highly correlated variables, so we fixed $\alpha =0.1$ which is often used for genetic association studies [30, 39].

If the *k*th phenotype outcome is qualitative with *K* levels, i.e., $y_{ik} \in \{1,2,\cdots ,K\}$ for all $i=1,\cdots ,n$, we can then replace the least square loss function by the negative logistic log-likelihood function

$$\begin{aligned} \sum _{i=1}^n \log \left( 1+e^{\beta _{0k}+{\varvec{x}}_i^{{\text{T}}}\varvec{\beta }_k}\right) - y_{ik}\left( \beta _{0k}+{\varvec{x}}_i^{{\text{T}}}\varvec{\beta }_k\right) \end{aligned}$$

for $K=2$ and the negative multinomial log-likelihood function for $K > 2$. Friedman et al. [38] described the explicit form of the multinomial likelihood function. For the sake of simplicity, we focus on $K=2$ for qualitative outcomes here.

In order to perform stable variable selection in regularization methods, Meinshausen and Bühlmann [40] proposed to compute the selection probability of individual variables. It does not require to choose the optimal $\lambda$, where cross-validation often fails to pick up a consistent value of $\lambda$ due to randomly splitting between training and validation sets. Selection probability essentially measures relative frequency of nonzero regression coefficients based on subsamples of the data. Shah and Samworth [41] adopted bootstrap resampling to compute the selection probability. If we denote the *l*th bootstrap sample by $b_l=\left\{ ({\varvec{x}}_1^*,{\varvec{y}}_1^*)^{[l]}, \ldots ,({\varvec{x}}_n^*,{\varvec{y}}_n^*)^{[l]}\right\}$, then the selection probability of the *j*th genetic variant and *k*th phenotype outcome is

$$\begin{aligned} s_{jk}(\lambda ) = \frac{1}{B} \sum _{l=1}^B I\left( {\hat{\beta }}_{jk}(b_l;\lambda ) \ne 0\right) , \end{aligned}$$

where $I(\cdot )$ is an indicator function and *B* is the total number of bootstrap resampling. For a pre-defined value of $\lambda$, the estimated regression coefficient ${\hat{\beta }}_{jk}(b_l;\lambda )$ can be computed by maximizing the penalized likelihood when we have the *l*th bootstrap sample. Friedman et al. [38] developed an R package ‘glmnet’ to provide the estimated regression coefficients of the elastic-net regularization for a grid of $\lambda$ values.

### Unified selection score

Let us denote the sum of the selection probabilities of the *k*th phenotype over *p* genetic variants by

$$\begin{aligned} S_k(\lambda )=\sum _{j=1}^p s_{jk}(\lambda ). \end{aligned}$$

Even though the *k*th phenotype outcome is not associated with any genetic variants, $S_k(\lambda ) > 0$ unless we put an extremely large value of $\lambda$. Elastic-net regularization was designed to perform variable selection, where variables with nonzero regression coefficients can be selected. But, one disadvantage is that it can choose any variables even when all variables are irrelevant. The number of nonzero regression coefficients generally increases as the numerical value of $\lambda$ decreases, regardless of association between a phenotype outcome and variants. In analysis of real genomic data, we cannot know how many phenotypes are truly associated with the genetic variants, and which phenotypes are associated with pleiotropic variants.

If there is no association with the *k*th phenotype outcome, nonzero regression coefficients of elastic-net regularization among *p* variants should be randomly occurred each bootstrap sample because they are all false positives. Consequently, $s_{jk}(\lambda )$ is expected to be uniformly distributed over *p* variants. In contrast, if the *k*th phenotype outcome is truly associated with the *j*th variant, $s_{jk}(\lambda ) > s_{j'k}(\lambda )$ for any $j'$th neutral variants so the distribution of selection probabilities is not uniform in this case. Therefore, a relatively large value of selection probability can be observed at only variant-associated phenotypes if the number of nonzero coefficients are all equal among *q* phenotypes, i.e., $S_1(\lambda _1)=S_2(\lambda _2)=\cdots =S_q(\lambda _q)$. However, it is almost impossible to figure out $\lambda _1,\ldots ,\lambda _q$ since bootstrap samples are random. Alternatively, we introduce new strategy for the sum of selection probabilities of *q* phenotypes to have the same quantity, while we weight genetic variants with a large selection probability.

For a pre-defined $\lambda$, let us denote the minimum value of $S_k(\lambda )$ among *q* phenotypes by $\eta _\lambda = \min _{1\le k\le q} S_k(\lambda )$. For the *k*th phenotype, we order *p* selection probabilities from largest to smallest such that

$$\begin{aligned} s_{[1], k}(\lambda ) \ge s_{[2], k}(\lambda ) \ge \cdots \ge s_{[p], k}(\lambda ). \end{aligned}$$

We define a threshold of selection probabilities of the *k*th phenotype by $\tau _k=s_{[J], k} (\lambda )$, where

$$\begin{aligned} J = \min \left\{ J'\in \{1,\ldots ,p\}: \sum _{j=1}^{J'} s_{[j], k}(\lambda ) \ge \eta _\lambda \right\} . \end{aligned}$$

The sum of the selection probabilities after thresholding is then

$$\begin{aligned} {\tilde{S}}_k(\lambda )=\sum _{j=1}^p s_{jk}(\lambda ) I\left( s_{jk} (\lambda ) \ge \tau _k \right) . \end{aligned}$$

Note that ${\tilde{S}}_k(\lambda )$ can be greater than $\eta _\lambda$ if there is a tie score among the *J*th largest selection probabilities. For instance, suppose that we have $s_{[1], k}(\lambda )=0.7$, $s_{[2], k}(\lambda )=0.2$, $s_{[3], k}(\lambda )=0.2$, $s_{[4], k}(\lambda )=0.1$ and $\eta _\lambda =1$. In this situation, $\tau _k=0.2$ and ${\tilde{S}}_k(\lambda )=1.1 > \eta _\lambda$. Therefore, we need to adjust individual selection probabilities so that ${\tilde{S}}_k(\lambda ) = \eta _\lambda$ for all $k=1,\ldots ,q$. In the above example, $s_{[1], k}^*(\lambda )=0.7/1.1$, $s_{[2], k}^*(\lambda )=0.2/1.1$ and $s_{[3], k}^*(\lambda )=0.2/1.1$ ensure for the sum of three adjusted selection probabilities to become $\eta _\lambda =1$. Although we chose the minimum value of $S_k(\lambda )$ for $k=1,\ldots , q$ to define $\eta _\lambda$, it can be defined in a different way such as a maximum value and a median value. If the maximum value is taken, i.e., $\eta _\lambda = \max _{1\le k\le q} S_k(\lambda )$, selection probabilities of $(q-1)$ phenotypes are upweighted. That is, selection probabilities of individual variants of the *k*th phenotype outcome are increased by $\eta _{\lambda }/S_k(\lambda )$ which is generally tiny for a large *p*. The key idea of thresholding is that the sum of adjusted selection probabilities of *q* phenotypes to have the same quantity. So, the adjusted selection probabilities of truly associated variants can be always higher than those of neutral variants among *q* phenotypes.

If the adjusted selection probability of the *j*th genetic variant and *k*th phenotype outcome is defined as

$$\begin{aligned} s_{jk}^*(\lambda ) = \frac{\eta _\lambda }{{\tilde{S}}_k(\lambda )} s_{jk}(\lambda )I\left( s_{jk} (\lambda ) \ge \tau _k \right) , \end{aligned}$$

the unified selection score of the *j*th variant is then

$$\begin{aligned} \pi _j (\lambda ) = \sum _{k=1}^q s_{jk}^*(\lambda ). \end{aligned}$$

For each genetic variant, the proposed unified selection score linearly combines the adjusted selection probabilities over *q* phenotypes. Finally, we can rank *p* variants based on their unified selection score. The selection score of variants that have a strong association with a single phenotype as well as moderate associations with multiple phenotypes tend to be high, while the selection score of variants with no association will be relatively low.

Finally, the tuning parameter $\lambda$ should be determined to compute our unified selection score. It essentially controls the number of nonzero regression coefficients, regardless of genetic association between variants and phenotype outcomes. If we employ a large value of $\lambda$, overall selection scores are too low to be compared with each other since large $\lambda$ leads to the small number of nonzero regression coefficients in elastic-net regularization. In contrast, an extremely small value of $\lambda$ results in too many nonzero coefficients, which requires explosive computational cost for the increasing number of coefficient estimates. An R package ‘glmnet’ [38], which estimates the coefficients of elastic-net regularization, generates 100 different $\lambda$ values such that

$$\begin{aligned} \lambda _{\max }=\lambda _1> \lambda _2> \ldots > \lambda _{100}=\lambda _{\min }, \end{aligned}$$

where $\lambda _{\max }$ is large enough to have ${\hat{\beta }}_j=0$ for all *j*, while $\lambda _{\min }$ produces the most number of nonzero coefficients. Our developed R package ‘UNISS’ chooses a median of 100 $\lambda$ values so that an appropriate number of nonzero coefficients can be estimated. Since the selection probability essentially measures relative selection frequency among *p* variables, the ranking of *p* variables based on our selection score are rarely changed along with a different value of $\lambda$ as discussed by Meinshausen and Bühlmann [40]. We also conducted a simulation study to compare selection performance of the proposed method for a different value of $\lambda$. We found that the median of 100 $\lambda$ values is adequate.

### A cut-off of unified selection scores

The unified selection score cannot select a certain number of genetic variants, but it prioritizes the *p* variants. So, we need to determine a cut-off value of the selection score in order to complete variable selection. A theoretical threshold of selection probabilities cannot be used in high-dimensional genomic data because of correlations among variables [40]. There have been some studies for an empirical threshold of selection probabilities [28, 42], but they are all limited to a single phenotype outcome. Computation of the empirical threshold is based on permutation, so we also employed it to find a cut-off value of our unified selection scores.

For an index set $I=\{1,2,\ldots ,n\}$, let us denote the *t*th permuted index set by $I_t=\{\sigma _t(1), \ldots , \sigma _t(n)\}$, where $\sigma _t(m)$ moves an index from place *m* to place $\sigma _t(m)$ in the *t*th permutation. Then, the *t*th permuted matrix of *q* phenotype outcomes can be written as

$$\begin{aligned} \begin{pmatrix} y_{\sigma _t(1)1} &{} y_{\sigma _t(1)2} &{} \ldots &{} y_{\sigma _t(1)q} \\ y_{\sigma _t(2)1} &{} y_{\sigma _t(2)2} &{} \ldots &{} y_{\sigma _t(2)q} \\ \vdots &{} \vdots &{} \ddots &{} \vdots \\ y_{\sigma _t(n)1} &{} y_{\sigma _t(n)2} &{} \ldots &{} y_{\sigma _t(n)q} \\ \end{pmatrix} \end{aligned}$$

Note that we conducted only row-wise permutation of the phenotype matrix and did not permute the genetic variants of ${\varvec{x}}_i$ for $i=1,\ldots ,n$. Therefore, permuted data basically assumes no genetic association between *q* phenotypes and *p* genetic variants.

The unified selection score of the *j*th variant based on the *t*th permutation set is then denoted by $\pi _j(I_t; \lambda )$, and they can be listed from largest to smallest such that

$$\begin{aligned} \pi _{[1]}(I_t; \lambda ) \ge \pi _{[2]}(I_t; \lambda ) \ge \cdots \ge \pi _{[p]}(I_t; \lambda ). \end{aligned}$$

Finally, the cut-off value of the unified selection scores is computed by

$$\begin{aligned} c_\lambda (\theta ) = \frac{1}{T} \sum _{t=1}^T \pi _{[\theta ]}(I_t; \lambda ), \end{aligned}$$

where *T* is a total number of permutation and $\theta$ is a pre-defined value representing the number of falsely selected variants. For a given value of $\lambda$ and $\theta$, the proposed method selects the variants $j\in \{1,\ldots ,p\}$, satisfying $\left\{ j: \pi _j(I; \lambda ) > c_\lambda (\theta ) \right\}$. Among the selected variants, the expected number of false positives should be around $\theta$ which plays a similar role as a significance level $\alpha$ in hypothesis testing. For example, if we choose $\theta =10$, the average value of the tenth largest selection scores over permutation sets is our cut-off value $c_\lambda (10)$. We then select a certain number of genetic variants whose unifiend selection scores are greater than $c_\lambda (10)$. Among these selected variants, the expected number of false positives should be around 10. Kim et al. [42] have demonstrated that the cut-off value of selection probabilities based on the quantile estimate of an empirical null distribution is able to control the number of false positives for a single phenotype outcome.

## Results

### Simulation studies

In order to generate single nucleotide polymorphism (SNP) data that has genetic correlations among adjacent variants, we first generated two *p*-dimensional vectors $(z_{i1, 1},\ldots ,z_{ip, 1})^{{\text{T}}}$ and $(z_{i1, 2},\ldots ,z_{ip, 2})^{{\text{T}}}$ from a multivariate normal distribution $N(0, \Sigma _x)$, where $\Sigma _x$ is an AR (1) covariance matrix with a correlation coefficient 0.9, i.e., the element at the *u*th row and *v*th column of $\Sigma _x$ is equal to $0.9^{|u-v|}$ for $1\le u,v \le p$. Note that AR(1) covariance produces highly correlated values for adjacent variables. Next, we generated a minor allele frequency (MAF) of the *j*th variant $\text {MAF}_j$ from an uniform distribution *U*(0.05, 0.5). If we define ${\bar{z}}_{ij, o}=\frac{1}{n} \sum _{i'=1}^n I(z_{ij, o} \le z_{i'j, o})$ for $o\in \{1, 2\}$, the genotype $x_{ij}\in \{0, 1, 2\}$ of the *i*-individual and the *j*th variant can be generated by

$$\begin{aligned} x_{ij} = I\left( {\bar{z}}_{ij, 1}< \text {MAF}_j\right) + I\left( {\bar{z}}_{ij, 2} < \text {MAF}_j\right) \end{aligned}$$

for $i=1,\ldots ,n$ and $j=1,\ldots ,p$. Using this procedure, the simulated SNP data can attain the Hardy-Weinberg equilibrium (HWE). It is a principle stating that the genetic variation in a population will remain constant from one generation to the next in the absence of disturbing factors [43]. In order to hold HWE, the genotype data should be generated based on their minor allele frequencies. For example, if the MAF of the *j*th SNP is denoted by $\text {MAF}_j$, then the probabilities of 3 different genotypes such as *AA*, *AC* and *CC* are $\text {MAF}_j^2$, $2\text {MAF}_j(1-\text {MAF}_j)$ and $(1-\text {MAF}_j)^2$, respectively. Since we hope that our simulation data is similar to real SNP data as much as possible, a particular data generation scheme was employed in order to hold HWE.

The phenotype outcome of the *i*th individual was then generated by

$$\begin{aligned} {\varvec{y}}_i = {\varvec{x}}_i^{{\text{T}}}\varvec{\beta }+ {\varvec{\epsilon }_i}, \end{aligned}$$

where an error vector ${\varvec{\epsilon }_i} = (\epsilon _{i1},\ldots ,\epsilon _{iq})^{{\text{T}}}$ follows a multivariate normal distribution $N(0, \Sigma _e)$. The error covariance $\Sigma _e$ has 1 on the diagonals and $\rho _e$ on the off-diagonals, where $\rho _e$ was generated from an uniform distribution *U*(0, 0.2) so that we can prevent the correlations among *q* phenotype outcomes being determined by only the error term. We observed that the sample correlation coefficient among *q* phenotype outcomes ranges from 0 to 0.9 with an average of 0.23. Note that the sample correlation coefficient among 4 quantitative traits from the our peanut data ranges from 0 to 0.531 with an average of 0.18. In our simulation, we fixed $n=400$, $p=40,000$ and $q=8$, which are comparable with our real SNP data.

Next, we located pleiotropic variants associated with paired phenotype outcomes, using nonzero regression coefficients. We assumed that pleiotropic variants are associated with only two phenotype outcomes. We randomly selected 16 disjoint regions where each region consists of 5 adjacent SNPs with either a homogeneous effect or a heterogeneous effect on two phenotype outcomes. For example, if the first and the second phenotypes are associated with the first 5 variants and their effects are homogeneous, then

$$\begin{aligned} \begin{array}{rcl} (\beta _{11}, \beta _{21}, \beta _{31}, \beta _{41}, \beta _{51}) &{} = &{} (0.5, 1.0, 1.5, 2.0, 2.5) \\ (\beta _{12}, \beta _{22}, \beta _{32}, \beta _{42}, \beta _{52}) &{} = &{} (0.5, 1.0, 1.5, 2.0, 2.5) \\ \end{array} \end{aligned}$$

If the effects of 5 variants are heterogeneous,

$$\begin{aligned} \begin{array}{rcl} (\beta _{11}, \beta _{21}, \beta _{31}, \beta _{41}, \beta _{51}) &{} = &{} (0.5, 1.0, 1.5, 2.0, 2.5) \\ (\beta _{12}, \beta _{22}, \beta _{32}, \beta _{42}, \beta _{52}) &{} = &{} (2.5, 2.0, 1.5, 1.0, 0.5) \\ \end{array} \end{aligned}$$

The first variant with a heterogeneous effect has a weak association signal with the first phenotype, i.e, $\beta _{11}=0.5$, while it has a strong association signal with the second phenotype, i.e., $\beta _{12}=2.5$. In contrast, the pleiotropic variants with a homogeneous effect have the same signal strength for the two phenotypes. We assumed that 8 regions have homogeneous effects while the other 8 regions have heterogeneous effects. If the *k*th phenotype is not associated with the *j*th variant, we simply set $\beta _{jk}=0$ and also all intercept parameters $\beta _{0k}=0$ for $k=1,\ldots ,8$.

For a qualitative outcome, we binarized a quantitative outcome based on its median value. Among 8 phenotype outcomes, we considered 6 quantitative and 2 binary $(Q, B) = (6, 2)$, 4 quantitative and 4 binary $(Q, B) = (4, 4)$ and 2 quantitative and 6 binary $(Q, B) = (2, 6)$. The number of phenotypes that have pleiotropic variants is either 4 (Half) or 8 (All). Therefore, there are a total number of 6 different scenarios in our simulation study. For each scenario, the selection performance of the proposed UNISS was compared with that of the 4 existing methods, which include MultiPhen [20] based on a reverse regression and 3 meta analysis methods. They are the minimum of *p*-values (MinP) [21], a meta-analysis version of USAT (metaUSAT) [23], and an adaptive test (AT) [25].

**Fig. 1:**
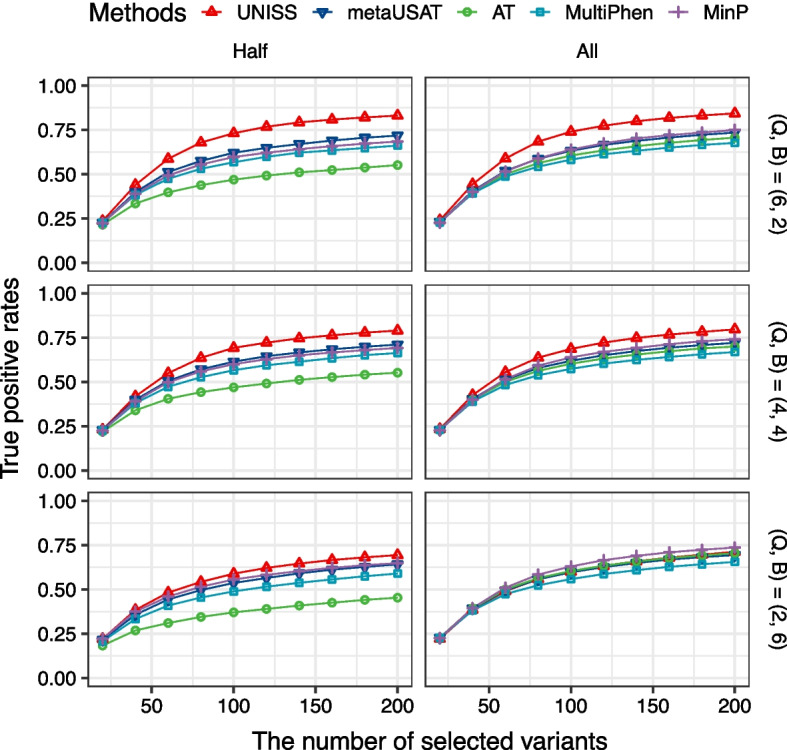
Averaged true positive rates of the top ranked 20, 40, $\ldots$, 200 variants selected by 5 statistical methods are displayed when the number of quantitative and binary phenotypes (Q, B) are (6, 2), (4, 4) or (2, 6), and the number of variant-associated phenotypes are either 4 (Half) or 8 (All)

Since only 80 pleiotropic variants among 40,000 have nonzero regression coefficients, the true positive rates (TPR) of 5 statistical methods were computed when each method selected the exactly same number of variants from 20 to 200 increased by 20. UNISS selected variants based on the ranking of the unified selection scores while the other 4 existing methods selected variants based on their *p*-value ranking of 40,000 variants. Figure 1 displays averaged true positive rates of 5 methods over 100 simulation replications in 6 different scenarios. It appears that the proposed selection method, UNISS has the highest TPR in 5 scenarios. In the last scenario where 2 quantitative and 6 binary phenotypes are all associated with pleiotropic variants, the TPR of UNISS is very close to TPRs of other methods. Also, it is noticeable that UNISS shows robust selection performance, regardless of the number of variant-associated phenotypes. In contrast, the TPR of AT was clearly dropped down when only half of phenotypes are associated with pleiotropic variants.

**Fig. 2:**
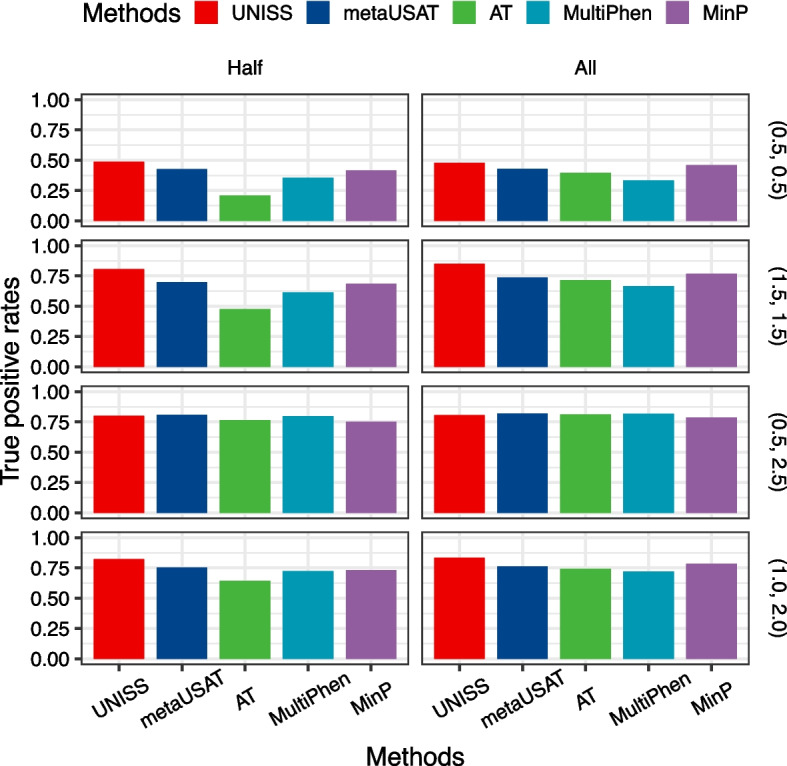
Averaged true positive rates of the top ranked 200 variants selected by 5 statistical methods are displayed when the pleiotropic variants have four different effect sizes such as (0.5, 0.5), (1.5, 1.5), (0.5, 2.5) and (1.0, 2.0), and the number of variant-associated phenotypes are either 4 (Half) or 8 (All)

Figure 2 shows the TPRs of 4 different pleiotropic variants when top 200 ranked variants were selected by each of 5 methods. UNISS has the highest TPR for selection of variants with a homogeneous effect such as (0.5, 0.5) and (1.5, 1.5). In contrast, AT has the lowest TPR when half of phenotypes are associated, while MultiPhen has the lowest TPR when all of phenotypes are associated. For pleiotropic variants with a heterogeneous effect of (0.5, 2.5), the selection performance of 5 methods are almost identical. Since the pleiotropic variant has a strong association signal with one phenotype outcome, it is easily detectable even if the association signal with the other phenotype outcome is very weak. However, for pleiotropic variants with a moderate effect size of (1.0, 2.0), the TPR of UNISS is clearly larger that TPRs of the other methods. Although pleiotropic variants with 3 different effect sizes of (1.5, 1.5), (0.5, 2.5) and (1.0, 2.0) have the same total effect of 3.0, four existing methods have quite different TPRs to detect the corresponding pleiotropic variants. In contrast to the exiting methods, UNISS shows consistent TPRs to find these variants.

**Fig. 3:**
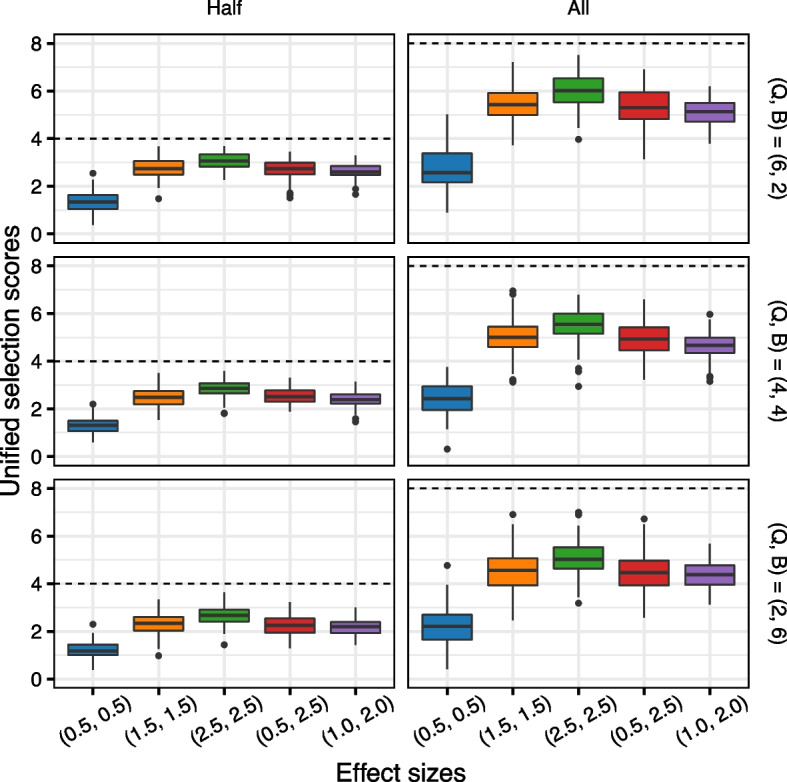
Box-plots of unified selection scores are displayed over 100 simulation replications when the pleiotropic variants have five different effect sizes such as (0.5, 0.5), (1.5, 1.5), (2.5, 2.5), (0.5, 2.5) and (1.0, 2.0), the number of quantitative and binary phenotypes (Q, B) are (6, 2), (4, 4) or (2, 6), and the number of variant-associated phenotypes are either 4 (Half) or 8 (All)

Next, we investigated the numerical values of unified selection scores when the pleiotropic variants have 5 different effect sizes in 6 different scenarios. Figure 3 shows the box-plots of unified selection scores over 100 simulation replications. It appears that selection scores are proportional to the effect sizes of pleiotropic variants. In addition, the selection score seems to approach the number of variant-associated phenotypes when the effect size of the variants increases. Although UNISS is not designed to identify which phenotype outcomes are associated with the pleiotropic variants, the selection scores are able to provide us with the expected number of variant-associated phenotype outcomes if association signals are strong enough.

Finally, we conducted a simulation study to control the number of false discoveries when we adopted the permutation cut-off of unified selection scores in order to select a certain number of variants. We focused on the scenario with 4 quantitative and 4 binary phenotypes $(Q, B) = (4, 4)$, where the number of variant-associated phenotypes is 8 (all). In Additional file 1, the number of true positives and the number of false discoveries were plotted each simulation replication when the expected number of falsely selected variants $\theta$ is given. The averaged number of selected variants over 100 simulation replications are 39.0, 49.4, 62.6, 72.7, 81.7 and 90.1 for $\theta =5, 10, 20, 30, 40$ and 50, respectively. We found that the number of false discoveries can be controlled well for only large $\theta$. We can see that the number of false discoveries was actually greater than $\theta$ in many simulation replications for small $\theta$. We considered another situation where genetic correlation of SNP data is moderate. So, we additionally generated SNP data using AR(1) covariance with a correlation coefficient of 0.6. In Additional file 2, we can see that the permutation cut-off of unified selection scores can control the number of false discoveries very well. Meinshausen and Bühlmann [40] have discussed the weakly independent assumption to control the number of false discoveries of selection probabilities. We also found that it is relatively difficult to control the number of false discoveries when genetic correlations are too high.

We additionally investigated the selection performance of the proposed method along with a different value of $\lambda$. For each simulation, we first generated 100 different $\lambda$ values from an R package ‘glmnet’ [38]. Next, we chose 9 different $\lambda$ values in order to apply the proposed selection method. They are from 0.1 to 0.9 quantiles of 100 $\lambda$ values, i.e., $\lambda _{10}> \lambda _{20}> \ldots > \lambda _{90}$ where $\lambda _{10}$ means the 0.9 quantile of 100 $\lambda$ values while $\lambda _{90}$ means the 0.1 quantile of 100 $\lambda$ values. Additional file 3 shows the TPRs of the proposed method along with different $\lambda$ values in 6 different scenarios. It appears that TPRs noticeably dropped down when a relatively large $\lambda$ is used. This is because the number of nonzero regression coefficients is too small to compute selection probability of individual variants, which can lead to inaccurate computation of unified selection scores. In contrast, TPRs are almost the same for all of $\lambda$ values from the median to the smallest. Computational cost which relies on the number of nonzero regression coefficient is increased as $\lambda$ decreases. Consequently, we chose the median of $\lambda$ for both accurate computation and computational efficiency.

### Real data analysis

In order to validate our proposed selection method in real data analysis, we applied it two different genotype datasets from cowpea and peanut crops. They were genotyped from the Illumina Cowpea iSelect consortium array and Axiom Arachis array, respectively. The cowpea dataset includes 49,683 SNPs with 384 samples, while the peanut dataset has 32,145 SNPs with 301 samples. The cowpea dataset has 6 phenotypes, including 2 quantitative and 4 binary traits [44]. 2 quantitative traits are pod length (PL) and seed numbers per pod (SNPP), while 4 binary traits are mature pod color (MPCOL), mature pod curve (MPCUV), seed density (SD) and shattering (ST). Each binary trait has the following categories: brown and black for MPCOL, straight and curved for MPCUV, low and high for SD, and shattering and no shattering for ST. The peanut dataset also has 6 phenotypes, including 4 quantitative and 2 binary traits [45]. 4 quantitative traits are leaf chlorophyll (LC), leaf aspect ratio (LAR), seed area (SA) and seed sucrose (SS), while 2 binary traits are flowering date (FD) and seed fungi quantity grade (SFQG), whose categories are early and late for FD and low and high for SFQG.

Before we identify pleiotropic variants of each dataset, we proceeded the quality control steps where we removed the samples with any missing phenotype values and then filtered out SNPs with either a missing call rate more than 70% or a minor allele frequency less than 1%. For remaining missing genotypes, we imputed the genotype values using an R package ‘synbreed’ [46], which imputes missing SNPs based on the posterior distribution of allele frequency. After the quality control steps, we ended up with 354 samples and 40,603 SNPs for the cowpea dataset and 235 samples and 27,991 SNPs for the peanut dataset.

We first investigated genetic correlations of each dataset to compare the correlations of simulated SNP data. Many crops including cowpea and peanut inherently have a complex population structure arising from historic inbreeding. This population structure often causes spurious estimation for linkage disequilibrium (LD). Alternatively, the partial correlation coefficient to correct bias induced by the population structure has been proposed [47, 48]. Therefore, we applied the partial correlation coefficients to two datasets for estimation of LD. For SNP data in our simulation studies, we generated it using AR(1) covariance with a correlation of $\rho _x=0.9$, but we also generated additional SNP data using 4 different correlations such as $\rho _x=0.5, 0.6, 0.7$ and 0.8. Additional file 4 displays the histogram of $\log _2 \text {LD}$ estimates for both cowpea and peanut datasets as well as 5 different simulated SNP data. Also, a sample mean and a standard deviation of $\log _2 \text {LD}$ estimates were computed for each dataset. It appears that the distribution of cowpea SNP data is similar to that of simulated SNP data using $\rho _x=0.9$. In contrast, the distribution of peanut data is close to that of simulated SNP data using $\rho _x=0.6$. Based on these histograms, we can conclude that the genetic correlations of cowpea dataset is quite strong, while the correlations of peanut dataset is moderate.

**Fig. 4:**
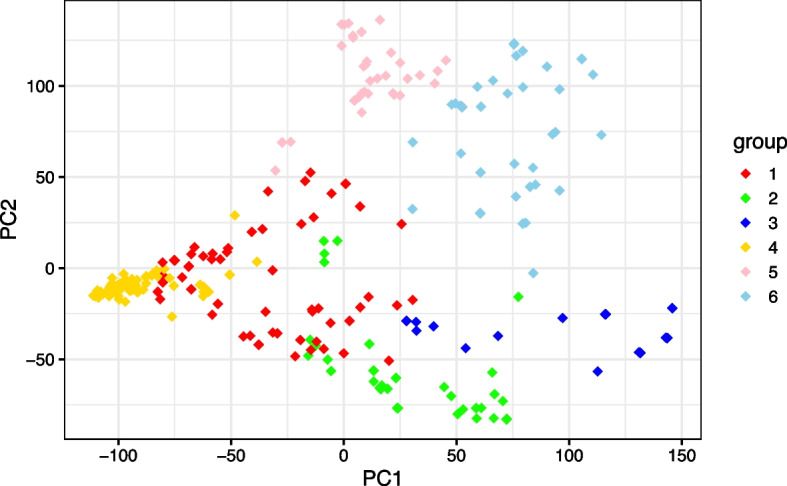
Scatter plot of the first two principal components colored by the population structure obtained from the *k*-means clustering for the cowpea dataset

Next, we conducted the *k*-means clustering to discover the population structure for both cowpea and peanut datasets. Since genomic data in crop plants are highly heterogeneous between populations, ignoring the population structure in genetic association studies may lead to spurious analysis results. The optimal number of clusters was computed by the gap statistic [49], where we found that both datasets have 6 clusters. The scatter plot of the first two principal components colored by the population structure are shown in Fig. 4 for the cowpea dataset and Additional file 5 for the peanut dataset. The index set of the population structure was then regarded as a covariate in analysis of pleiotropic variants, because we assume that it can affect the phenotype outcomes. Note that a covariate is not penalized in the elastic-net regularization, so it always has a nonzero regression coefficient, regardless of the tuning parameter $\lambda$. The penalized likelihood function including covariates is described in Friedman et al. [38].

In order to detect pleiotropic variants, we did not apply only the proposed UNISS, but also 3 meta analysis methods such as MinP, AT and metaUSAT used in our simulation study. For cowpea dataset, the number of variants selected by UNISS was 40, 46, and 68 for $\theta = 30, 40$ and 50, respectively. MinP selected 21, 41, and 105 variants for the significance level of $10^{-12}$, $10^{-11}$ and $10^{-10}$, respectively. For the same significance levels, AT selected 46, 70, and 88 variants, while metaUSAT selected 73, 85, and 118 variants, respectively. If we apply Bonferroni adjustment for a level of 0.05, the variants with their *p*-values less than $1.2314\times 10^{-6}$ are significant, where 3 meta analysis methods selected hundreds of variants with this level. Since selection of too many variants can drastically increase the number of false positives, we just focused on top 20 ranked variants for fair comparison of four methods including UNISS, MinP, AT and metaUSAT. Similar to our simulation study, UNISS selected top 20 variants based on their selection scores, while three meta analysis methods selected 20 variants based on their *p*-value rankings.

**Fig. 5:**
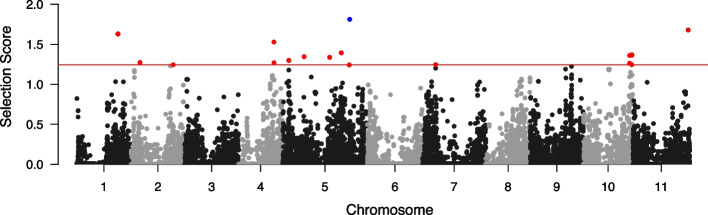
Manhattan plot of the unified selection scores for the cowpea dataset. Top 20 ranked variants uniquely identified by UNISS are colored by red and variants commonly identified by three meta analysis methods are colored by blue

A venn diagram summarizing the top 20 variants selected by each of 4 methods is shown in Additional file 6. Surprisingly, only 1 variant is commonly selected by 4 methods. 19 variants were identified by only UNISS, while 3 variants were identified by only MinP. There are no variants uniquely identified by either AT or metaUSAT. 16 variants were commonly selected by 3 meta analysis methods. Additional file 7 shows information of the selected variants, their unified selection scores and their *p*-values of 3 meta analysis methods. Also, we conducted a univariate test for these variants, where a generalized linear model was applied using a single trait on the response and both a variant and a covariate on the predictors. The variant $\text {rs}\_2\_23708$ on chromosome 3 was identified by all of four methods since the pod length trait is strongly associated with it. UNISS also has the largest selection score for this variant. Among the 19 variants identified by only UNISS, we can see that most of variants have either moderate or strong association with at least one of 6 traits. However, we should notice 3 variants including $\text {rs}\_2\_35607$ on chromosome 3, $\text {rs}\_2\_49550$ on chromosome 10, and $\text {rs}\_2\_37568$ on chromosome 5. Their *p*-values of the univariate test are not small enough to detect significant association. That is, single-trait analyses fail to identify these variants. Correspondingly, the *p*-values of 3 meta analysis methods are also relatively large for these variants. We can conclude that these three variants are potentially pleiotropic variants that are weakly associated with multiple traits. The manhattan plot of the cowpea dataset are displayed in Fig. 5, where the unified selection scores were replaced by $-\log _{10}$ *p*-values.

In the same way, we also identified top 20 ranked variants of the peanut dataset using four methods. Additional file 8 shows a venn diagram of four methods for top 20 ranked variants. We can see that 7 variants are selected by all of 4 methods, while UNISS uniquely identified 13 variants. Also, we can see information of the selected variants, their unified selection scores and their *p*-values of 3 meta analysis methods in Additional file 9. Similar to the cowpea dataset, 7 variants commonly identified by four methods have extremely small *p*-values of the univariate test for seed area trait. Therefore, these variants are easily detectable by single-trait analyses. However, among 13 variants identified by only UNISS, 3 variants such as AX-176823712 on chromosome Araip.B07, AX-176819477 on chromosome Aradu.A09, and AX-176811925 on chromosome Aradu.A04 have relatively large *p*-values of the univariate tests. So, they cannot be detected by single-trait analyses. We also think that these variants should be further investigated as potentially pleiotropic variants. Additional file 10 displays the manhattan plot of the cowpea dataset.

## Conclusions

We have proposed a new selection method to identify pleiotropic variants associated with both quantitative and qualitative traits. Specifically, we proposed unified selection scores, one score for one variant and then ranked them from largest to smallest. We also proposed a procedure to select a cut-off value of the unified selection scores to select a certain number of variants while controlling the number of false discoveries. In our simulation studies, we have demonstrated that the proposed selection method has higher true positive rates than the existing meta analysis methods when effect sizes of pleiotropic variants are relatively small and genetic variants are highly correlated with each other. In real data applications to two plant SNP datasets with the cowpea and peanut datasets, we have shown that our method can identify potentially pleiotropic variants weakly associated with multiple traits, which cannot be detected by single-trait analyses.

Although unified selection scores are computed based on coefficient estimates of an elastic-net regularization, we can easily extend to other regularization methods. For example, sparse group lasso regularization combines the lasso penalty and the group lasso penalty, encouraging a group effect of variants [50]. Network-based regularization utilizes genetic network information such as gene regulatory networks and protein-protein interaction networks into genetic association studies. It has been demonstrated that using genetic networks in association studies can improve true positive selection [51–53]. Since the unified selection score essentially combines the number of nonzero regression coefficients of individual variants, it can be directly extended to other regularization methods.

Our method was applied to both quantitative and binary traits from two plant SNP datasets in this article. For other types of traits, we can simply replace the likelihood part of the penalized regression. For instance, a conditional logistic likelihood function can be used for 1:1 matched case–control outcome [30]. Also, a partial Cox likelihood function can be replaced for a survival time outcome [54]. Therefore, the proposed unified selection score is flexible and can be readily extended to other types of traits other than quantitative and qualitative traits we focused in this work. Additionally, the proposed method is not limited to analysis of SNP data. Since the elastic-net regularization method can estimate the regression coefficients of quantitative predictors, the unified selection score can be applied to different kinds of genomic data such as gene expression data and DNA methylation data.

Note that the current method is computationally intensive due to bootstrap and permutation procedures. Bootstrap sampling is necessary to compute selection probabilities and permutation procedures are to find a cut-off value of the unified selection scores. Although this is feasible with current computation technology, new computational strategy should be developed to further reduce the computational cost. In our analysis with the peanut dataset, UNISS requires around 428 s to compute the unified selection scores of 27,991 variants for a single $\lambda$. Similarly, it takes approximately 986 s for UNISS to compute the selection scores of 40,603 variants from the cowpea dataset. The machine we used for computational time is Intel(R) Xeon(R) E5-2640 v4 @ 2.40GHz processor with 128 GB memory.

### Supplementary Information

**Additional file 1**. For simulated SNP data with a genetic correlation of 0.9, the number of true positives and the number of false discoveries are plotted each of 100 simulation replications, when the expected number of falsely selected variants $\theta$ is fixed as 5, 10, 20, 30, 40 and 50.

**Additional file 2**. For simulated SNP data with a genetic correlation of 0.6, the number of true positives and the number of false discoveries are plotted each of 100 simulation replications, when the expected number of falsely selected variants $\theta$ is fixed as 5, 10, 20, 30, 40 and 50.

**Additional file 3**. Averaged true positive rates of the proposed method are displayed along with 9 different λ values that are chosen from 0.1 to 0.9 quantiles of 100 λ values when the number of quantitative and binary phenotypes (Q, B) are (6, 2), (4, 4) or (2, 6), and the number of variant-associated phenotypes are either 4 (Half) or 8 (All).

**Additional file 4**. Histograms of log_2_ LD estimates of cowpea data, peanut data and simulated SNP data using 5 different correlations ρ_x_ = 0.5, 0.6, 0.7, 0.8 and 0.9 are shown with the sample mean ± the standard deviation of log_2_ LD estimates for each data.

**Additional file 5**. Scatter plot of the first two principal components colored by the population structure obtained from the k-means clustering for the peanut dataset.

**Additional file 6**. Venn diagram summarizing the top 20 SNPs ranked by UNISS, MinP, AT and metaUSAT for the cowpea dataset.

**Additional file 7**. Among the top 20 variants of the cowpea dataset selected by each of UNISS, MinP, AT and metaUSAT, the unified selection scores of UNISS and the *p*-values of MinP, AT and metaUSAT are shown for the variants (a) commonly identified by four methods, and (b) uniquely identified by UNISS. The *p*-values of univariate test were computed by a generalized linear model.

**Additional file 8**. Venn diagram summarizing the top 20 SNPs ranked by UNISS, MinP, AT and metaUSAT for the peanut dataset.

**Additional file 9**. Among the top 20 variants of the peanut dataset selected by each of UNISS, MinP, AT and metaUSAT, the unified selection scores of UNISS and the *p*-values of MinP, AT and metaUSAT are shown for the variants (a) commonly identified by four methods, and (b) uniquely identified by UNISS. The *p*-values of univariate test were computed by a generalized linear model.

**Additional file 10**. Manhattan plot of the unified selection scores for the peanut dataset. Top 20 ranked variants uniquely identified by UNISS are colored by red and variants commonly identified by three meta analysis methods are colored by blue.

## Data Availability

An R package ‘UNISS’ can be downloaded from http://github.com/statpng/uniss. The datasets generated and analysed during the current study are available in the European Variation Archive (EVA) at EMBL-EBI under accession number PRJEB59561 for the cowpea dataset and under accession number PRJEB60506 for the peanut dataset.
