# Supplementary material for: New statistical selection method for pleiotropic variants associated with both quantitative and qualitative traits
Source: BMC Bioinformatics. 2023 Oct 10;24:381. doi: 10.1186/s12859-023-05505-8 (PMC10563219; doi:10.1186/s12859-023-05505-8)
Supplement: Supplementary file 1 — Additional file 1. For simulated SNP data with a genetic correlation of 0.9, the number of true positives and the number of false discoveries are plotted each of 100 simulation replications, when the expected number of falsely selected variants $\theta$θ is fixed as 5, 10, 20, 30, 40 and 50. [file 12859_2023_5505_MOESM1_ESM.pdf]

Additional file 1

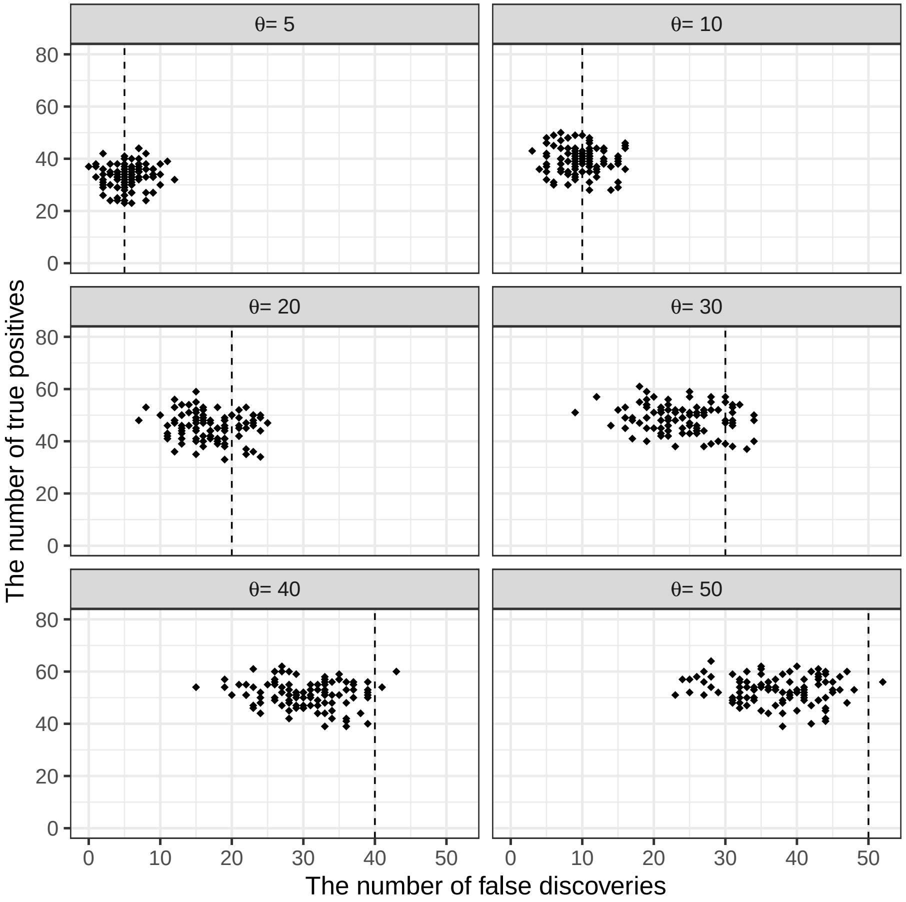

For simulated SNP data with a genetic correlation of 0.9, the number of true positives and the number of false discoveries are plotted each of 100 simulation replications, when the expected number of falsely selected variants θ is fixed as 5, 10, 20, 30, 40 and 50.
