# Supplementary material for: New statistical selection method for pleiotropic variants associated with both quantitative and qualitative traits
Source: BMC Bioinformatics. 2023 Oct 10;24:381. doi: 10.1186/s12859-023-05505-8 (PMC10563219; doi:10.1186/s12859-023-05505-8)
Supplement: Supplementary file 3 — Additional file 3. Averaged true positive rates of the proposed method are displayed along with 9 different λ values that are chosen from 0.1 to 0.9 quantiles of 100 λ values when the number of quantitative and binary phenotypes (Q, B) are (6, 2), (4, 4) or (2, 6), and the number of variant-associated phenotypes are either 4 (Half) or 8 (All). [file 12859_2023_5505_MOESM3_ESM.pdf]

Additional file 3

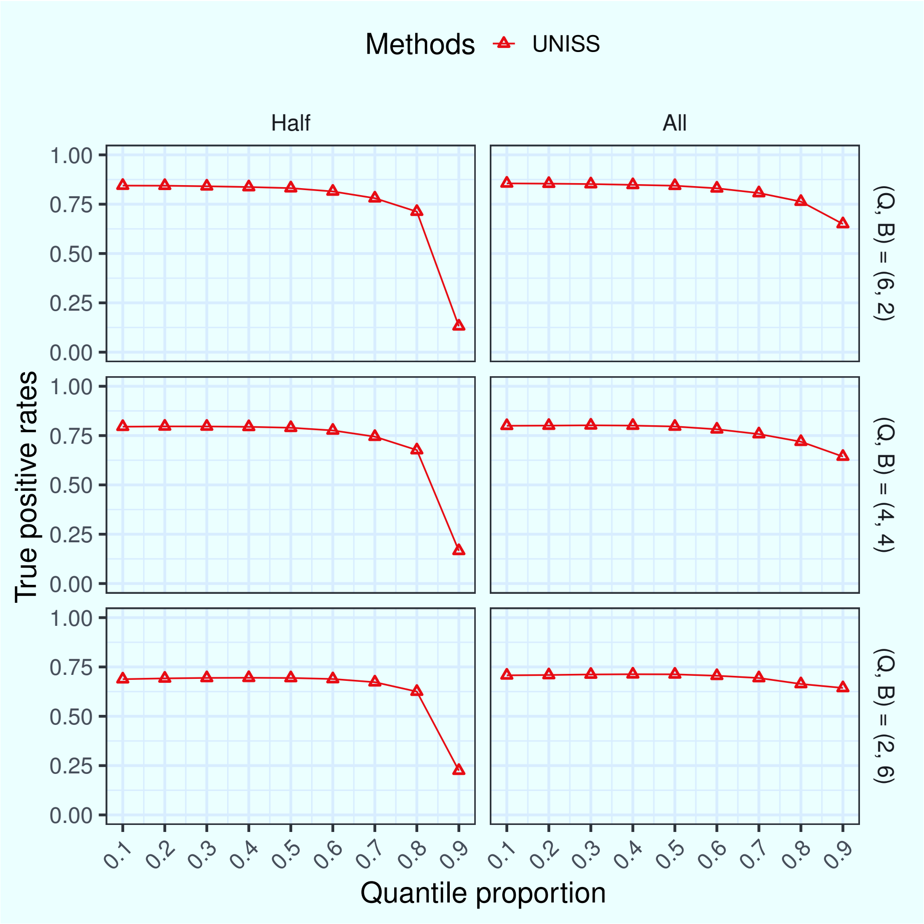

Averaged true positive rates of the proposed method are displayed along with 9 different λ values that are chosen from 0.1 to 0.9 quantiles of 100 λ values when the number of quantitative and binary phenotypes (Q, B) are (6, 2), (4, 4) or (2, 6), and the number of variant-associated phenotypes are either 4 (Half) or 8 (All).
