# Supplementary material for: New statistical selection method for pleiotropic variants associated with both quantitative and qualitative traits
Source: BMC Bioinformatics. 2023 Oct 10;24:381. doi: 10.1186/s12859-023-05505-8 (PMC10563219; doi:10.1186/s12859-023-05505-8)
Supplement: Supplementary file 4 — Additional file 4. Histograms of log2 LD estimates of cowpea data, peanut data and simulated SNP data using 5 different correlations ρx = 0.5, 0.6, 0.7, 0.8 and 0.9 are shown with the sample mean ± the standard deviation of log2 LD estimates for each data. [file 12859_2023_5505_MOESM4_ESM.pdf]

Additional file 4

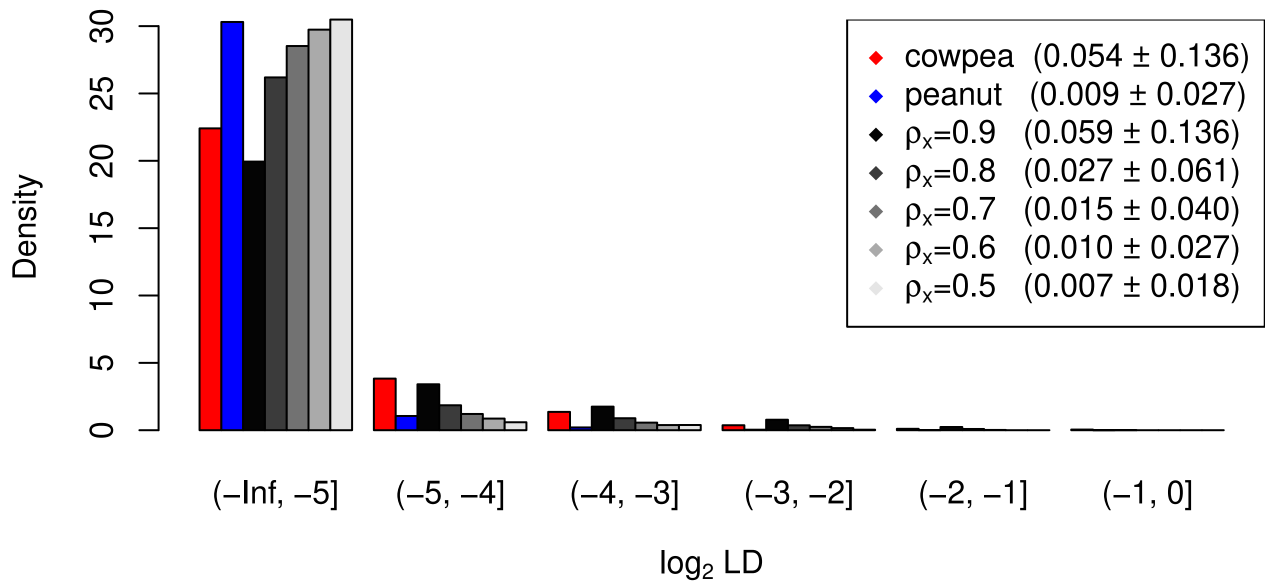

Histograms of $\log_2$ LD estimates of cowpea data, peanut data and simulated SNP data using 5 different correlations $\rho_x = 0.5, 0.6, 0.7, 0.8$ and 0.9 are shown with the sample mean $\pm$ the standard deviation of $\log_2$ LD estimates for each data.
