# Supplementary material for: New statistical selection method for pleiotropic variants associated with both quantitative and qualitative traits
Source: BMC Bioinformatics. 2023 Oct 10;24:381. doi: 10.1186/s12859-023-05505-8 (PMC10563219; doi:10.1186/s12859-023-05505-8)
Supplement: Supplementary file 6 — Additional file 6. Venn diagram summarizing the top 20 SNPs ranked by UNISS, MinP, AT and metaUSAT for the cowpea dataset. [file 12859_2023_5505_MOESM6_ESM.pdf]

Additional file 6

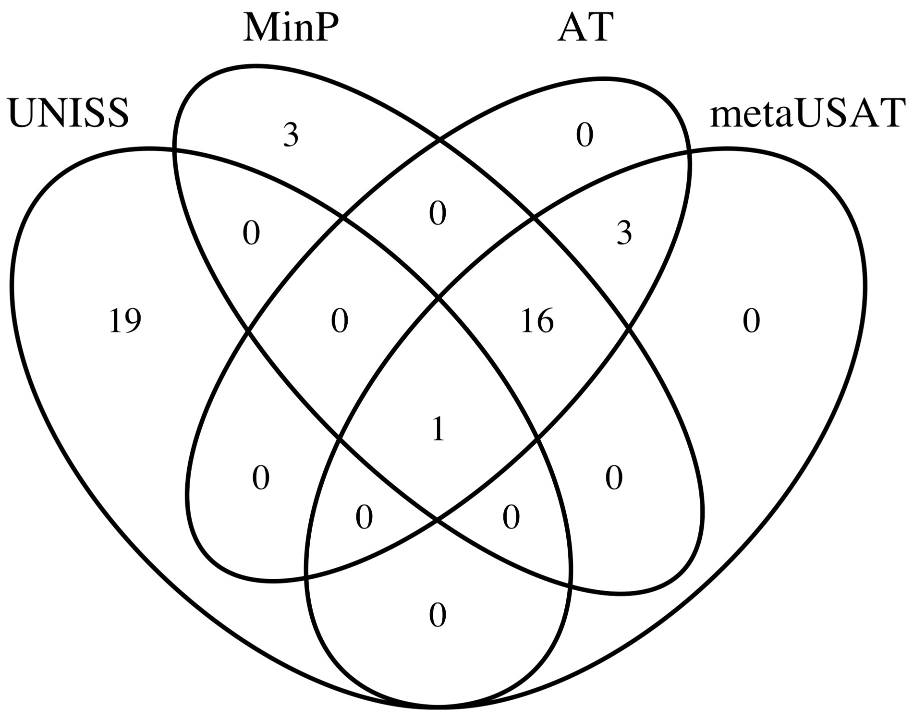

Venn diagram summarizing the top 20 SNPs ranked by UNISS, MinP, AT and metaUSAT for the cowpea dataset.
