# Supplementary material for: New statistical selection method for pleiotropic variants associated with both quantitative and qualitative traits
Source: BMC Bioinformatics. 2023 Oct 10;24:381. doi: 10.1186/s12859-023-05505-8 (PMC10563219; doi:10.1186/s12859-023-05505-8)
Supplement: Supplementary file 7 — Additional file 7. Among the top 20 variants of the cowpea dataset selected by each of UNISS, MinP, AT and metaUSAT, the unified selection scores of UNISS and the p-values of MinP, AT and metaUSAT are shown for the variants (a) commonly identified by four methods, and (b) uniquely identified by UNISS. The p-values of univariate test were computed by a generalized linear model. [file 12859_2023_5505_MOESM7_ESM.pdf]

Additional file 7

Among the top 20 variants of the cowpea dataset selected by each of UNISS, MinP, AT and metaUSAT, the unified selection scores of UNISS and the p-values of MinP, AT and metaUSAT are shown for the variants (a) commonly identified by four methods, and (b) uniquely identified by UNISS. The p-values of univariate test were computed by a generalized linear model.

(a) 1 variant commonly identified by four methods

rs	chrom	pos	UNISS	MinP	AT	metaUSAT	Univariate test					
							MPCOL*	MPCUV*	SD*	ST*	PL	SNPP
rs_2_23708	3	51714447	1.8123	<1e-16	4.0e-36	2.2e-39	0.4950	0.2843	0.3074	0.9804	6.0e-20	0.9043

(b) 19 variants uniquely identified by UNISS

rs	chrom	pos	UNISS	MinP	AT	metaUSAT	Univariate test					
							MPCOL*	MPCUV*	SD*	ST*	PL	SNPP
rs_2_16525	9	42650146	1.6780	2.3e-08	2.1e-06	2.5e-07	0.5674	0.0150	0.1777	7.1e-05	1.0e-08	0.8461
rs_2_00097	1	31921605	1.6298	8.0e-09	3.0e-08	1.6e-09	0.0543	0.0023	0.3521	1.8e-07	3.9e-09	0.8071
rs_2_23119	1	31925122	1.6298	8.9e-09	3.0e-08	1.6e-09	0.0543	0.0023	0.3521	1.8e-07	3.9e-09	0.8071
rs_2_35678	2	27213173	1.5276	3.8e-10	1.3e-08	4.4e-09	0.7380	0.0493	0.9785	8.7e-05	2.7e-10	0.3268
rs_2_51910	3	45265789	1.3925	2.5e-10	1.3e-08	3.1e-09	0.3340	0.0172	0.1927	8.9e-05	1.8e-10	0.9508
rs_2_27927	8	37461449	1.3697	8.8e-08	6.0e-06	3.2e-07	0.2337	0.0007	0.9537	0.0001	3.1e-08	0.9317
rs_2_09764	8	37448628	1.3626	4.3e-07	2.7e-05	4.7e-07	0.0620	0.0002	0.6614	0.0006	1.5e-07	0.8915
rs_2_14432	8	35744524	1.3601	6.2e-12	4.0e-13	2.2e-14	0.0191	0.1870	0.8396	0.0004	7.9e-12	0.5004
rs_2_35607	3	16546703	1.3454	0.1498	0.5188	0.5733	0.9977	0.9871	0.9843	0.4707	0.4211	0.0293
rs_2_44978	3	36291935	1.3371	4.3e-10	2.1e-09	7.1e-10	0.0039	0.0430	0.6504	0.0029	2.6e-10	0.1439
rs_2_10631	3	4812132	1.2988	3.4e-06	4.6e-08	7.9e-09	0.7299	0.0018	1.9e-05	0.0230	0.0026	1.0e-06
rs_2_15004	3	4827812	1.2988	3.7e-06	4.6e-08	7.9e-09	0.7299	0.0018	1.9e-05	0.0230	0.0026	1.0e-06
rs_2_49550	10	6840319	1.2729	0.09972	0.02060	0.0210	0.0185	0.1729	0.0789	0.3573	0.6877	0.3972
rs_2_36064	2	27256798	1.2701	2.4e-09	7.6e-08	2.1e-08	0.6639	0.0389	0.8939	7.4e-05	1.3e-09	0.2578
rs_2_14431	8	35746941	1.2635	2.8e-11	3.2e-12	2.1e-13	0.0230	0.2083	0.7953	0.0006	3.0e-11	0.5840
rs_2_37568	5	10083010	1.2458	0.0024	0.0003	0.0002	0.0004	0.0068	0.3730	0.8044	0.0019	0.0400
rs_2_23299	8	37447290	1.2453	1.1e-06	5.5e-05	9.2e-07	0.0704	0.0002	0.6922	0.0007	3.3e-07	0.8317
rs_2_17152	10	32484024	1.2438	0.0001	2.3e-07	4.0e-08	0.7956	0.0007	9.2e-05	0.0897	0.0002	2.9e-05
rs_2_32085	3	51441205	1.2421	2.0e-09	1.4e-10	6.9e-11	0.4191	0.9118	0.1233	0.0215	1.2e-09	0.4872

MPCOL: mature pod color; MPCUV: mature pod curve, SD: seed density; ST: shattering; PL: pod length; SNPP: seed numbers per pod.

rs: Reference SNP (rs) number; chr: chromosome; pos: chromosomal position

Asterisk (*) indicates a binary trait.
