# Supplementary material for: New statistical selection method for pleiotropic variants associated with both quantitative and qualitative traits
Source: BMC Bioinformatics. 2023 Oct 10;24:381. doi: 10.1186/s12859-023-05505-8 (PMC10563219; doi:10.1186/s12859-023-05505-8)
Supplement: Supplementary file 8 — Additional file 8. Venn diagram summarizing the top 20 SNPs ranked by UNISS, MinP, AT and metaUSAT for the peanut dataset. [file 12859_2023_5505_MOESM8_ESM.pdf]

Additional file 8

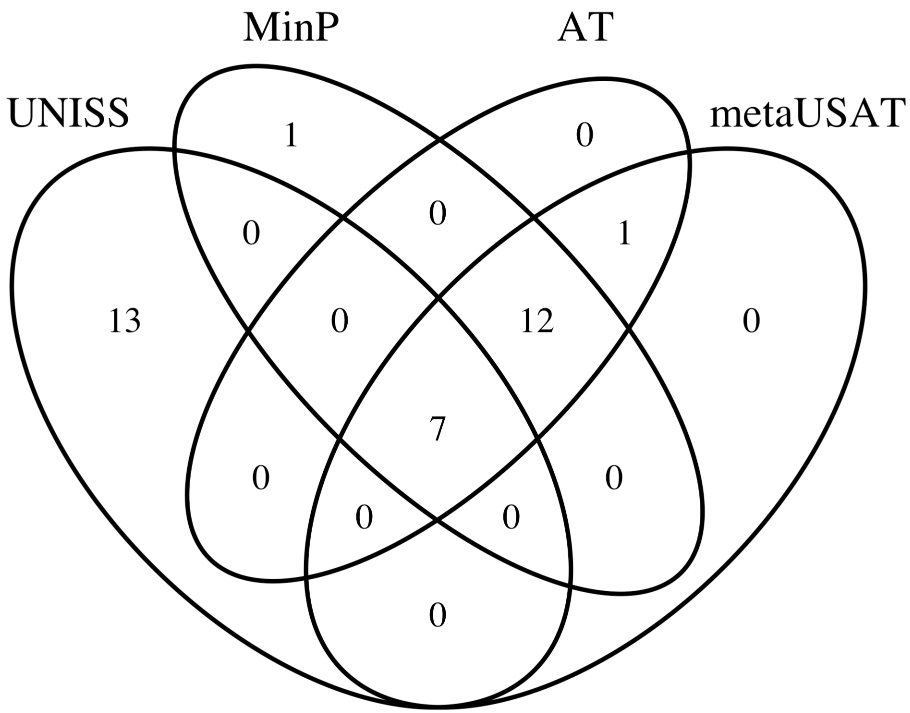

Venn diagram summarizing the top 20 SNPs ranked by UNISS, MinP, AT and metaUSAT for the peanut dataset.
