# Supplementary material for: New statistical selection method for pleiotropic variants associated with both quantitative and qualitative traits
Source: BMC Bioinformatics. 2023 Oct 10;24:381. doi: 10.1186/s12859-023-05505-8 (PMC10563219; doi:10.1186/s12859-023-05505-8)
Supplement: Supplementary file 9 — Additional file 9. Among the top 20 variants of the peanut dataset selected by each of UNISS, MinP, AT and metaUSAT, the unified selection scores of UNISS and the p-values of MinP, AT and metaUSAT are shown for the variants (a) commonly identified by four methods, and (b) uniquely identified by UNISS. The p-values of univariate test were computed by a generalized linear model. [file 12859_2023_5505_MOESM9_ESM.pdf]

Additional file 9

Among the top 20 variants of the peanut dataset selected by each of UNISS, MinP, AT and metaUSAT, the unified selection scores of UNISS and the p-values of MinP, AT and metaUSAT are shown for the variants (a) commonly identified by four methods, and (b) uniquely identified by UNISS. The p-values of univariate test were computed by a generalized linear model.

(a) 7 variants commonly identified by four methods

rs	chrom	pos	UNISS	MinP	AT	metaUSAT	Univariate test					
							LC	LAR	SA	SS	FD*	SFQG*
AX-147253833	Araip.B06	129731047	1.31789	<1e-16	3.5e-27	3.4e-30	0.0015	2.8e-05	1.4e-23	0.7983	0.0144	0.7948
AX-147253998	Araip.B06	132403515	1.219721	<1e-16	3.7e-25	6.0e-28	0.0532	2.0e-06	2.5e-21	0.7663	0.0176	0.1985
AX-147226313	Aradu.A06	105285553	1.176701	<1e-16	3.0e-20	1.6e-22	0.0195	0.00145	1.8e-18	0.6579	0.1210	0.8954
AX-176823393	Araip.B06	128311575	1.149215	<1e-16	4.1e-35	5.5e-39	0.2034	3.6e-06	1.3e-26	0.2076	0.0014	0.0753
AX-176802785	Araip.B06	109240201	1.113031	<1e-16	2.3e-23	5.8e-26	0.0502	5.3e-07	4.5e-20	0.6883	0.0738	0.7388
AX-176807953	Aradu.A09	113907685	1.087346	<1e-16	4.2e-27	4.1e-30	0.0369	4.6e-05	3.4e-23	0.0216	0.0004	0.9348
AX-147262340	Araip.B09	143554366	1.067092	<1e-16	1.5e-21	6.1e-24	0.5937	9.1e-06	2.1e-18	0.0335	0.0004	0.5314

(b) 13 variants uniquely identified by UNISS

rs	chrom	pos	UNISS	MinP	AT	metaUSAT	Univariate test					
							LC	LAR	SA	SS	FD*	SFQG*
AX-176795320	Araip.B03	9128023	1.4556	4.6e-13	8.6e-11	4.7e-13	1.5e-06	0.0097	2.1e-12	0.0302	0.0041	0.6190
AX-177639817	Aradu.A07	62373156	1.4029	2.4e-13	2.1e-15	4.0e-17	0.0064	0.3280	1.0e-12	2.4e-05	0.9840	0.2890
AX-176815650	Aradu.A03	48229	1.3631	4.1e-13	2.2e-14	5.3e-16	0.0002	0.8770	1.9e-12	0.0018	0.9840	0.1330
AX-147243136	Araip.B03	3096124	1.3244	5.3e-08	5.5e-08	5.8e-09	5.7e-07	0.7380	3.3e-08	0.0207	0.0095	0.8610
AX-176813024	Araip.B06	129725307	1.3018	<1e-16	4.6e-15	3.4e-17	0.0023	0.0003	1.8e-16	0.1380	0.0085	0.1300
AX-176813277	Aradu.A06	106448614	1.2092	1.7e-14	1.0e-15	2.0e-17	0.1930	2.9e-07	1.3e-13	0.4200	0.5940	0.7930
AX-176806809	Araip.B04	105619478	1.1875	<1e-16	1.2e-14	7.6e-17	6.5e-05	0.0065	3.7e-16	0.0217	0.0017	0.8350
AX-176791829	Aradu.A06	110928524	1.1509	0.0001	5.1e-05	3.6e-05	0.3710	0.0040	0.3510	2.4e-05	0.1910	0.4230
AX-176823712	Araip.B07	122814564	1.0530	0.0063	6.4e-05	4.4e-05	0.1230	0.0677	0.0250	0.0013	0.8010	0.0412
AX-176819477	Aradu.A09	120303150	1.0446	0.0045	6.5e-05	4.5e-05	0.2620	0.6660	0.1210	0.0009	0.0069	0.2230
AX-177637358	Araip.B09	12912661	1.0281	4.5e-12	4.7e-11	2.5e-12	0.0530	0.5360	1.1e-11	0.2390	0.1230	0.0946
AX-176799159	Aradu.A04	105153999	1.0144	4.4e-07	2.6e-07	2.3e-07	2.1e-05	0.3400	1.8e-07	0.5590	0.9870	0.8580
AX-176811925	Aradu.A04	9573590	1.0138	0.0058	0.0078	0.0050	0.5740	0.0925	0.0869	0.0135	0.7910	0.0010

LC: leaf chlorophyll; LAR: leaf aspect ratio, SA: seed area; SS: seed sucrose; FD: flowering date; SFQG: seed fungi quantity grade.

rs: Reference SNP (rs) number; chr: chromosome; pos: chromosomal position

Asterisk (*) indicates a binary trait.
