# Supplementary material for: New statistical selection method for pleiotropic variants associated with both quantitative and qualitative traits
Source: BMC Bioinformatics. 2023 Oct 10;24:381. doi: 10.1186/s12859-023-05505-8 (PMC10563219; doi:10.1186/s12859-023-05505-8)
Supplement: Supplementary file 10 — Additional file 10. Manhattan plot of the unified selection scores for the peanut dataset. Top 20 ranked variants uniquely identified by UNISS are colored by red and variants commonly identified by three meta analysis methods are colored by blue. [file 12859_2023_5505_MOESM10_ESM.pdf]

Additional file 10

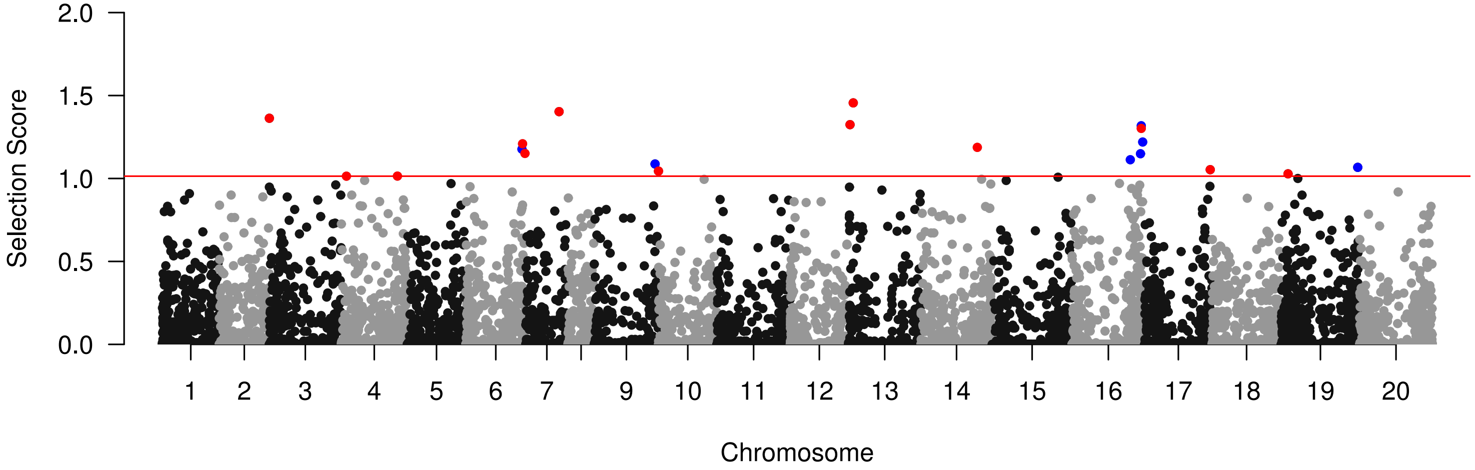

Manhattan plot of the unified selection scores for the peanut dataset. Top 20 ranked variants uniquely identified by UNISS are colored by red and variants commonly identified by three meta analysis methods are colored by blue.
